# Primary mediastinal choriocarcinoma in an 18‐year‐old male with pulmonary and brain metastasis: A case report

**DOI:** 10.1111/crj.13691

**Published:** 2023-08-23

**Authors:** Hanlin Wang, Xiu Chen, Renquan Zhang

**Affiliations:** ^1^ Department of Thoracic Surgery The First Affiliated Hospital of Anhui Medical University Hefei China

**Keywords:** choriocarcinoma, chorionic gonadotropin, male, mediastinal, multiple metastases

## Abstract

Primary mediastinal choriocarcinoma, also known as non‐pregnant choriocarcinoma, is a rare malignancy unrelated to pregnancy, with a higher incidence in males. And primary mediastinal choriocarcinoma is mostly associated with organ and lymph node metastasis, with rapid progression and poor prognosis. Here, we report an extremely rare case of the primary anterior mediastinal choriocarcinoma that occurred in an 18‐year‐old man with multiple metastases of the lung and brain.

## Graphical abstract

This was an extremely rare case of the primary anterior mediastinal choriocarcinoma that occurred in an 18‐year‐old man with multiple metastases of the lung and brain. At present, there are no effective treatment options for primary mediastinal choriocarcinoma. The patient in this case used a conventional EP protocol (etoposide, cisplatin). We hope to provide valuable experience in treating such patients.

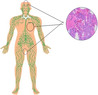

## 1 INTRODUCTION

Primary mediastinal choriocarcinoma is a rare malignancy unrelated to pregnancy. The main atypical symptoms are cough, hemoptysis, headache, and so forth. Primary mediastinal choriocarcinoma has atypical symptoms and is mostly advanced when symptoms appear, making early diagnosis difficult for pathologists.^1^ Although some patients can achieve partial remission after multiple chemotherapy regimens and radiotherapy, drug resistance and disease progression often result in an extremely poor prognosis.^2^ Clinically, there are few reports on primary choriocarcinoma. This article reports a case of primary mediastinal choriocarcinoma in a man and analyzes it based on the relevant literature. We hope to deepen the understanding of primary mediastinal choriocarcinoma, improve the early diagnosis of this disease, and provide experience for clinical treatment.

## 2 CASE REPORT

**FIGURE 1:**
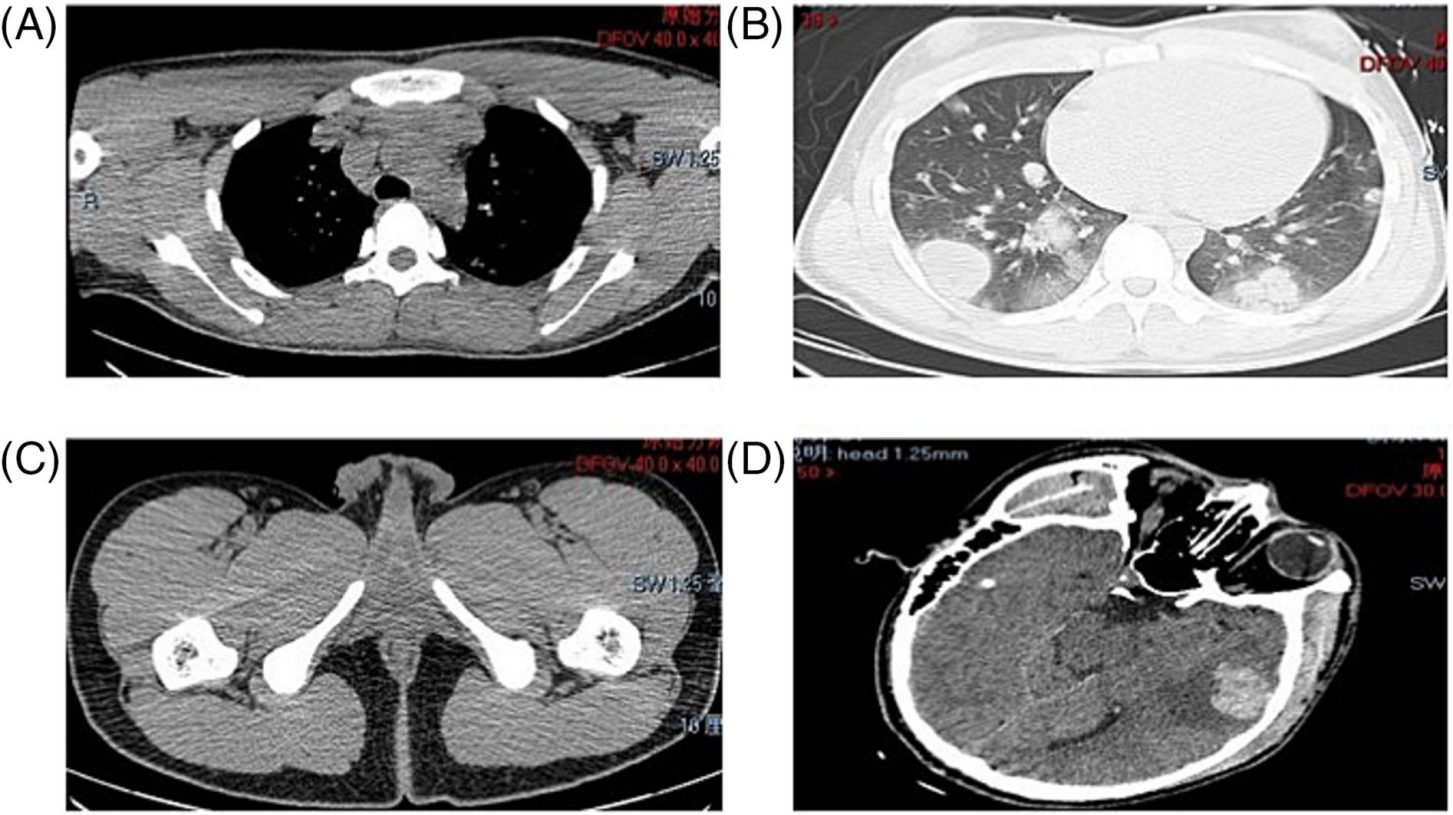
Total body CT scan. (A) A mediastinal window image revealed a heterogeneous tumor. (B) A pulmonary window image showed masses in the both lung lobe. (C) Pelvis CT showed no abnormal mass shadow in the bladder or gonads. (D) Left frontotemporal brain hemorrhage.

**FIGURE 2:**
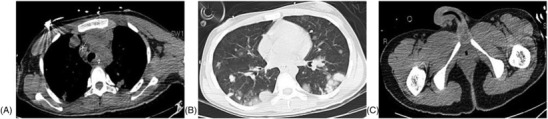
Total body CT scan. (A and B) The mediastinal masses and multiple nodules in both lungs did not shrink significantly. (C) Pelvis CT showed no abnormal mass shadow as before.

**FIGURE 3:**
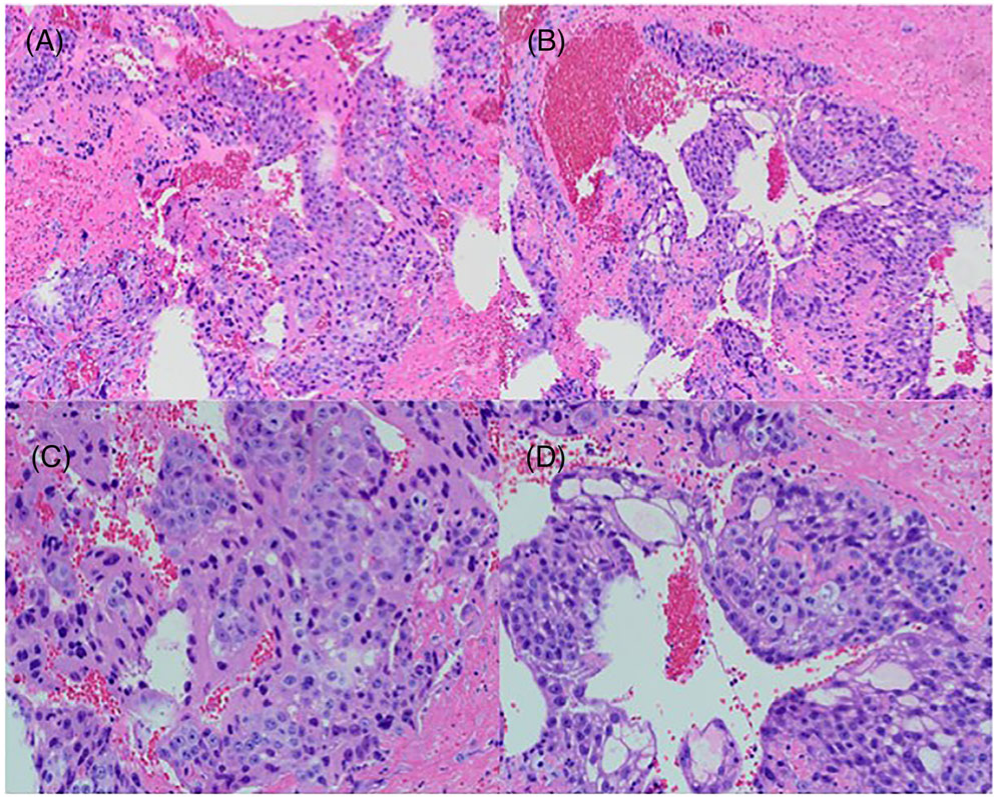
Surgical specimen of the brain. (A and B) Histological examination shows that the tumor consists of two types of trophoblasts, with cytotrophoblasts growing in clusters separated by syncytial trophoblasts (hematoxylin and eosin staining, magnification, ×100). (C and D) Hematoxylin and eosin staining, magnification, ×200.

An 18‐year‐old teenager was treated at the First Affiliated Hospital of Anhui Medical University for a sudden headache for 2 days before admission. The patient had no specific history of disease, family history, and history of psychological disorders and had undergone facial cystectomy 1 year ago, yet the detailed pathological finding was not known. The patient was a student and had no history of smoking or secondhand smoking and no history of headache. The patient had a temperature of 36.2°C, a heart rate of 69 beats, a blood pressure of 121/71 mmHg, a height of 180 cm, respiratory rate of 18, and a weight of 70 kg. Physical examination revealed that the patient was confused and mentally irritable, with a Glasgow Coma Scale (GSC) score of 14 (medical assessment of a patient's level of coma). Emergency total body CT scan suggested left frontotemporal brain hemorrhage, the anterior mediastinal lesions, and multiple nodules on both lungs (Figure 1). Due to the patient's agitated state, other special examination could not be safely refined. To alleviate the urgent symptoms, the surgery of temporal lobe lesion excision was performed on the fourth day after admission. Intraoperatively, the tumor was located in the lateral fissure, dark brown, mixed with old blood clots. And the solid part of the tumor was rotten fish‐like, flesh‐red, and rich in blood supply and contained a large number of lesioned vessels. There were no accidents during the operation, and there was little intraoperative bleeding. The patient returned safely to the ward after the operation, and the lesion was sent for pathological diagnosis. Accidentally, the day after the surgery, the patient had a sudden hemoptysis (24‐h hemoptysis volume greater than 300 mL), accompanied by poor oxygenation, and was in critical condition (respiratory rate 40 times per minute, blood oxygen saturation 90%, and heart rate 97 times per minute); thus, he was transferred to the intensive care medicine department. Serum chorionic gonadotropin examination showed the ultrahigh levels of chorionic gonadotropin (β‐HCG 1490890 mIU/mL, normal 0–5 mIU/mL). Pathology confirmed primary choriocarcinoma measured 5.0 cm × 5.0 cm × 2.0 cm, also known as non‐pregnant choriocarcinoma. Due to the poor condition of the patient, we were unable to perform a puncture biopsy to confirm the primary lesion. Based on imaging evidence and clinical symptoms of the patient, as well as extensive literature references, we identified the case as the anterior mediastinum choriocarcinoma. According to multi‐disciplinary team (MDT),^3^ because of the patient's poor condition, radiotherapy and thoracic surgery were not recommended. Finally, resuscitation treatment was prioritized in the intensive care medicine department, and chemotherapy was administered after stabilization of base conditions. However, the prognosis was not good. Considering that it was less than half a month after the craniotomy, unconscious and in poor general physical condition, priority was given to a relatively mild EP protocol (etoposide and cisplatin, day 1–day 5, etoposide 120 mg, cisplatin 35 mg). During this process, the patient developed myelosuppression, resulting in extremely low levels of white blood cells. Then he was given leukocyte‐raising treatment. After a cycle of treatment, the level of chorionic gonadotropin (β‐HCG) in the serum significantly decreased (β‐HCG 36851 mIU/mL, normal 0–5 mIU/mL). However, the mediastinal masses and multiple nodules in both lungs did not shrink significantly (Figure 2). Total body CT scan shows the anterior mediastinal mass and multiple nodules in both lungs. The patient was treated in the intensive care unit for approximately 4 weeks. During the period, he received a series of symptomatic treatment such as dehydration, reduction of cranial pressure, anti‐infection, protection of organ function, nutritional support, and tracheotomy. To treat the patient's hemoptysis, we performed fiberoptic bronchoscopy to stop the hemoptysis, which was effective and relieved the patient's symptoms. And we did not perform further angiography. After one cycle of chemotherapy, pulmonary bleeding gradually stopped, oxygenation improved, and basic conditions were stable. Subsequently, the patient was transferred back to the general ward for following treatment (respiratory rate 26 times per minute, blood oxygen saturation 98.7%, and heart rate 78 times per minute). Before the second cycle of chemotherapy, the patient was discharged. The last measured serum chorionic gonadotropic hormone (β‐HCG) before discharge was 4638 mIU/mL. The pathological result of the surgical specimen identified the diagnosis of metastatic choriocarcinoma (Figure 3). Further IHC staining indicated that the tumor cells were positive for HCG‐B (+), HPL (+), GPC‐3 (+), Inhibin‐a (+), EMA (+), SALL4 (+), P63 (+), GATA‐3 (+), P53 (+, 40%), and Ki‐67 (+, 70%). And EP protocol (etoposide and cisplatin, day 1–day 5, etoposide 120 mg, cisplatin 35 mg) was adopted. The patient was followed up 1 month after his discharge from the hospital and had stable vital signs. The patient is currently preparing for her second chemotherapy treatment. Three months later, we once again followed up via telephone. The patient has completed approximately six cycles of chemotherapy. A review of the CT scan indicates a substantial reduction in the lesions within both lungs, and β‐HCG levels have significantly fallen to normal ranges. During this process, due to diminished immunity, the patient has developed varicella of an infectious nature and is currently receiving treatment. Regrettably, due to certain limiting factors, subsequent cycles of the patient's chemotherapy were carried out at a local hospital. Consequently, we were unable to acquire more accurate radiological data and a precise chemotherapy regimen.

## 3 DISCUSSION

Choriocarcinoma is classified as primary or secondary choriocarcinoma. Secondary choriocarcinoma often occurs in women of childbearing age. The disorder is characterized by elevated β‐HCG and Chorionic cells in the tissue. Primary choriocarcinoma is a rare malignancy unrelated to pregnancy, with a higher incidence in men.^4^ Primary mediastinal choriocarcinoma is particularly rare, with fewer than 50 cases reported from 1931 when it was first reported until 2023. Primary mediastinal choriocarcinoma needs to be differentiated from bronchopulmonary carcinoma, lymphoma, mediastinal cyst, and thymoma. Clinical symptoms are atypical, including chest pain, cough, hemoptysis, shortness of breath, and weakness, often combined with multiple pulmonary metastases.^5^ The patient in this article also presented with pulmonary and brain metastases. Laboratory tests usually show high levels of β‐hCG, which can be an important diagnostic and prognostic marker with a high specificity and sensitivity.^6^ Unlike the gestational choriocarcinoma, primary choriocarcinoma has a poor prognosis in most cases, and most cases have extensive metastases at the time of diagnosis, with a short survival period.^7^ In this case, there was no obvious evidence of primary genital tumor, CT scan suggested the possibility of multiple metastases in both lungs, and intracranial surgical pathology confirmed the metastases, so we diagnosed as primary mediastinal choriocarcinoma. Female choriocarcinoma is sensitive to chemotherapy with a better prognosis.^8^ In contrast, there is no standardized chemotherapy regimen for male patients, and the high‐intensity chemotherapy regimen is likewise used for men.^9^ Because of the poor condition of this patient, thoracic surgery could not be performed. The patient was treated with an EP induction regimen (etoposide and cisplatin), but as observed in this case, after one course of treatment, the mediastinal and bilateral lung lesions did not shrink. It is possible that the course of treatment is not enough.

Programmed cell death ligand 1 (PD‐L1) inhibitor is a popular alternative therapy in recent years, and PD‐L1 are commonly expressed in choriocarcinoma tumors.^10^ Pan and Hou^11^ reported a case treated with pembrolizumab after an ineffective induction of EP alone. After two cycles, β‐hCG levels dropped dramatically but soon increased again. The mediastinal tumor shrank after using pembrolizumab, but the disease also deteriorated quickly.

We searched for patients with primary mediastinal choriocarcinoma from PubMed for nearly two decades (Table 1). We found that the primary mediastinal choriocarcinoma was mostly in the anterior mediastinum, and the survival of these patients was basically no more than 6 months. The treatment for primary mediastinal choriocarcinoma also varied.

**TABLE 1:** Studies on primary mediastinal choriocarcinoma.

First author	Reference	Year	Age	Sex	Location	Treatment	Result	Overall survival
Chen Han	^9^	2020	26	M	Mediastinal	EMA/CO, TP	Died	6.5 months
Zheng Ruan	^12^	2012	28	M	Mediastinal	Chemotherapy	NR	More than 24 months
Takashi Yuri	^13^	2006	19	M	Mediastinal	NR	Died	About 3 days
Ivo M. B. Francischetti	^14^	2017	41	M	Mediastinal	VIP	NR	NR
Weiyu Pan	^11^	2022	19	M	Mediastinal	EP, radiotherapy, pembrolizumab, EMA/CO	Died	4 months
Hua‐hao Shen	^15^	2004	19	M	Mediastinal	NR	Died	2 months
Khaled A. Murshed	^16^	2021	25	M	Mediastinal	EP, VIP, TIP, EMA/CO	Died	6 months
Zhenhua Qiu	^7^	2019	26	M	Mediastinal	EMA/CO, TP	Died	6.5 months
Tadashi Sakane	^17^	2020	26	M	Mediastinal	Bleomycin, cisplatin, and etoposide	Died	About 9 days
Suat‐Jin Lu	^18^	2013	22	M	Mediastinal	VIP	NR	NR
Weigang Zhao	^1^	2019	40	M	Mediastinal	EP	Died	15 months
Juliane Bachmann	^19^	2007	31	F	Mediastinal	Chemotherapy	NR	NR
I. Kuno	^20^	2016	58	F	Mediastinal	EMA/CO, radiotherapy	Died	41 days
E.J. Yun	^21^	2005	45	F	Mediastinal	Chemotherapy	Died	2 weeks

Abbreviations: EMA/CO, actinomycin D, etoposide, methotrexate, cyclophosphamide, Vincristine; EP, etoposide, cisplatin; NR, not reported; TP, cisplatin, paclitaxel; VIP, etoposide, cisplatin, ifosfamide.

In summary, there is no established standardized treatment protocol for primary mediastinal choriocarcinoma in men. Cytotoxic chemotherapy remains the mainstay of treatment, although most patients develop resistance to chemotherapy and die from progression. Emerging immune checkpoint inhibitors (ICIs) may be a potential measure for patients with primary choriocarcinoma, but the exact effect is still unclear. Large‐scale studies of primary choriocarcinoma are less likely to be conducted because such diseases are extremely rare. We hope the case report will provide valuable experience for the treatment of this disease.

## AUTHOR CONTRIBUTIONS

All authors contributed to the conception and drafting of the manuscript. Hanlin Wang and Xiu Chen analyzed the data.

## CONFLICT OF INTEREST STATEMENT

The authors have no conflicts of interest to declare.

## ETHICS STATEMENT

The authors are accountable for all aspects of the work in ensuring that questions related to the accuracy or integrity of any part of the work are appropriately investigated and resolved. All procedures performed in this study were in accordance with the ethical standards of the institutional and/or national research committee(s) and with the Helsinki Declaration (as revised in 2013). Written informed consent was obtained from the patient for publication of this case report and accompanying images. A copy of the written consent is available for review by the editorial office of this journal.

## Data Availability

The data that support the findings of this study are available from the corresponding author upon reasonable request.
